# Medical Opinion and Movement

**Published:** 1909-03-06

**Authors:** 


### Medical Opinion and Movement.

"Winter vomiting" is the designation given by Dr. N. E. Kouchev, of Saratov, to a morbid affection he has observed in five cases, in which daily vomiting, especially in the morning, has occurred for several years through the winter months without any evidence of organic disease to account for the condition. Of these five patients, four were women of ages varying from 27 to 60, the fifth being a man aged 36. All had suffered from the trouble for several years; in the case of the woman, aged 32, for ten years. The vomiting generally occurred in the morning before breakfast and during the day some time after meals. The vomit was normal and showed no signs of mucus or blood. In certain cases there was some nausea and colic pain, but in all other respects the patients were normal. They became weak and emaciated, but completely recovered health in the warmer weather. Analysis of the gastric contents showed in one case some hyper-acidity, and in one case deficient acid and pepsin secretion, so that the vomiting appeared to be quite independent of the quantity or quality of gastric secretion. Dr. Kouchev compares the condition to "winter hæmoglobinuria" and "winter prurigo" described by other authors, and thinks that the pathology of the affection is to be sought in the nervous system. Under the influence of the cold the peripheral nerve-endings in the skin, and perhaps also in the mucous membranes, are irritated, and this irritation is transmitted reflexly to the nerves of the stomach.

---

The therapeutic value of fibrolysin injections as a means of causing absorption of cicatricial tissue may be said to be still *sub judice*. By many it has been tried, found wanting, and discarded. On the other hand, favourable accounts still appear from time to time of its use in certain conditions. Recently it was reported in these columns as having given successful results in cases of spinal sclerosis. In the last issue of *Guy's Hospital Gazette* Mr. W. M. Mollison gives an account of some cases of deafness resulting from a fibrosis of the auditory apparatus (adhesions following otitis media, fibrosis of the auditory ossicles, sclerosis of the membrana tympani, etc.), in which the effect of injections of fibrilysin has been tried. Merck's fibrolysin in ampullæ containing 2.3 c.c. each was used and injected into the gluteal region three times a week for several weeks. Of five cases so treated, two were distinctly benefited, two showed some improvement, but one of them is quite recent and still under treatment; one case was apparently unaffected, although the patient said she heard better. Although the results are not altogether convincing, they appear to justify a more extended use of the treatment in such cases. Schnütgen advocates this line of treatment in cases of pleural adhesions following effusion, and in the *Berliner Klinische Wochenschrift* he reports on several cases in which he has obtained good effects by injections of fibrolysin either locally or into the gluteal muscles once or twice a week.

---

The much-vexed question of shock and the condition of the peripheral vessels during shock, have formed the object of a series of experiments by Drs. Seelig and Lyon, the results of which appear in an article in the *Journal of the American Medical Association*. They studied visually the retinal vessels and measured the outflow from the temporal vein in dogs. They found that the proportional rate of flow from the vein during shock was more rapid after division of the sciatic nerve, showing that the section of the nerve caused vaso-dilatation of the vessels of the leg, just as it does in an unshocked animal. This dilatation was, however, proportionately greater than it is in a normal animal, and the difference is accounted for on the assumption that in the animal in the condition of shock, before division of the sciatic nerve, the vessels are more contracted than they are in a normal animal. The suggestion is, therefore, that in shock the peripheral vessels are actually more contracted than under normal conditions. Observations on the retinal vessels gave the same indications. The fundi of the dog's eyes were examined before and after the shock, and the size of the vessels carefully noted. In every instance it is stated that the vessels showed a marked degree of contraction after the animal was in shock.

---

The study of the phenomena of hæmolysis and agglutination has already demonstrated many interesting facts, such as the relative hæmolytic power of different sera for the corpuscles of different species of animals, the hæmolysis being greater as the species are more widely related. Further research on these lines has shown that hæmolysis may prove of value in the diagnosis of disease. In certain diseases the serum may show an autolytic effect on the corpuscles of the same individual, or the serum in certain diseases, notably cancer, may agglutinate the corpuscles of normal individuals while the corpuscles of other individuals affected with the same disease resist this action. A good deal of work has been carried out by Dr. R. Weil He experimented on dogs affected with transplantable lympho-sarcoma, and found that fresh extracts from the undegenerated tumours were lytic to the corpuscles of normal dogs, and still more so extracts from necrosed portions of the tumours. The sera, too, were more hæmolytic than those of normal dogs, and the corpuscles of the tumour dogs were more refractory to hæmolysis than the normal. Observations on cancer patients showed that in about 50 per cent. of cases the serum was hæmolytic to the corpuscles of others, but not auto-hæmolytic. Crile, employing Weil's method, has obtained even more striking results. He finds that in nearly all early cases of cancer the serum is hæmolytic, and the corpuscles are resistant to their own or other cancer serum. In a recent paper he reports observations on the blood of 600 patients. He obtained hæmolysis in 48 out of 52 cases of tuberculosis, and in 130 out of 153 cancer cases. Those of tuberculosis, however, showed reverse hæmolysis, *i.e.*, the patients' corpuscles were hæmolytic to normal serum.

